# Supplementary material for: The encapsulin from Thermotoga maritima is a flavoprotein with a symmetry matched ferritin-like cargo protein
Source: Sci Rep. 2021 Nov 23;11:22810. doi: 10.1038/s41598-021-01932-w (PMC8610991; doi:10.1038/s41598-021-01932-w)
Supplement: Supplementary file 1 — Supplementary Information. [file 41598_2021_1932_MOESM1_ESM.pdf]

Supplementary Information for:

The encapsulin from *Thermotoga maritima* is a flavoprotein with a symmetry matched ferritin-like cargo protein

Benjamin J. LaFrance,[1,ⱡ] Caleb Cassidy-Amstutz,[1,ⱡ] Robert J. Nichols,[1] Luke M. Oltrogge,[1] Eva Nogales,[1,2,3] David F. Savage[1*]

[1] Department of Molecular and Cell Biology, University of California Berkeley, Berkeley, CA, USA.
[2] Howard Hughes Medical Institute, University of California, Berkeley, CA, USA.
[3] Molecular Biophysics and Integrated Bioimaging Division, Lawrence Berkeley National Laboratory, Berkeley, CA, USA.
[ⱡ]these authors contributed equally to this work
[*]savage@berkeley.edu

The Supplementary Information file contains:

Figs. S1 to S6
Table S1

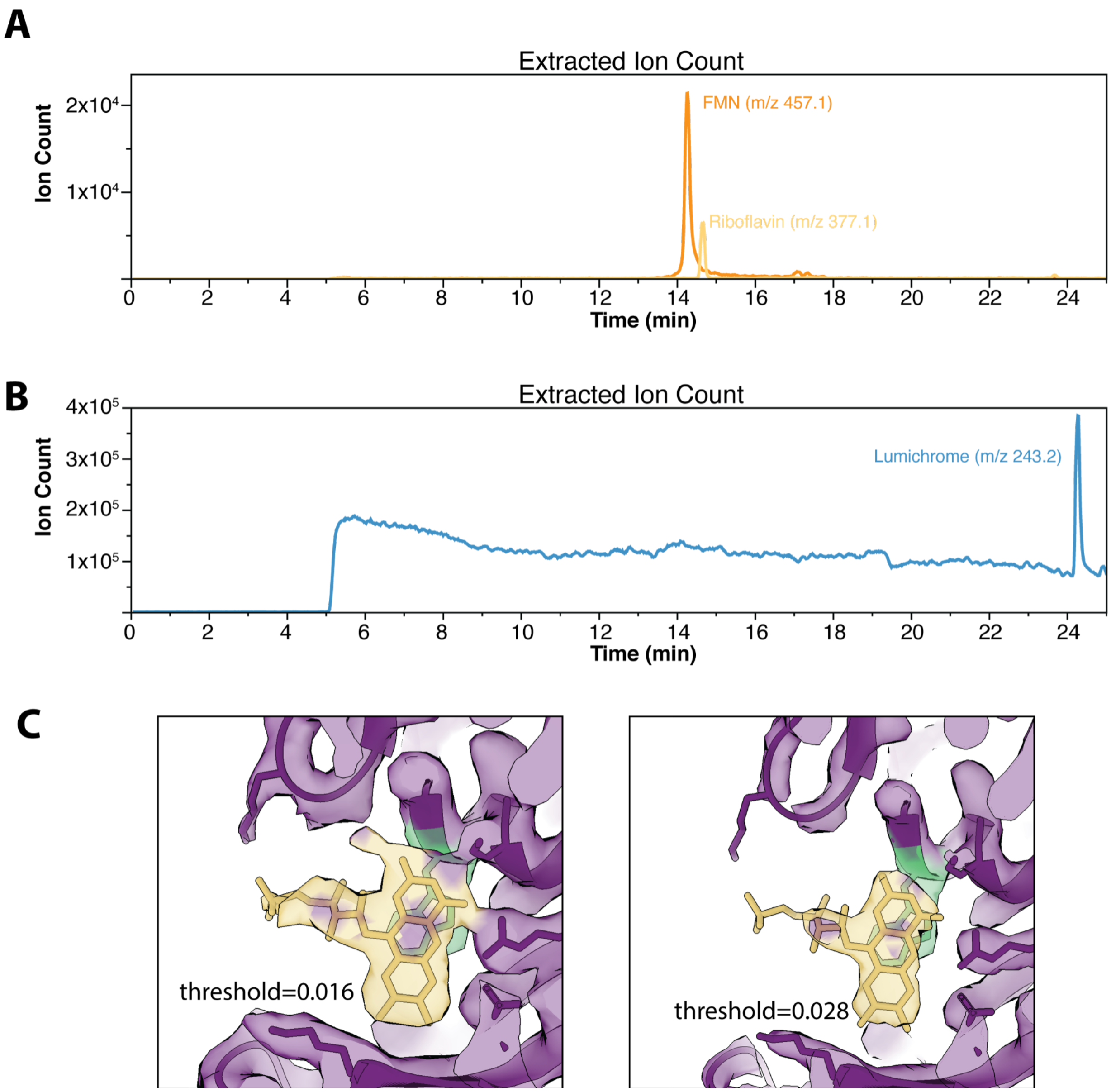

Fig. S1. Mass spectrometry confirms flavin presence in the *T. maritima* encapsulin. (A) and **(B)** Extracted ion counts show that FMN (orange) riboflavin (yellow), and lumichrome (blue) are all present within a pure *T. maritima* encapsulin sample. **(C)** CryoEM density of the flavin at two different thresholds showing density for the FMN and riboflavin R-group that is absent in the lumichrome degradation product.

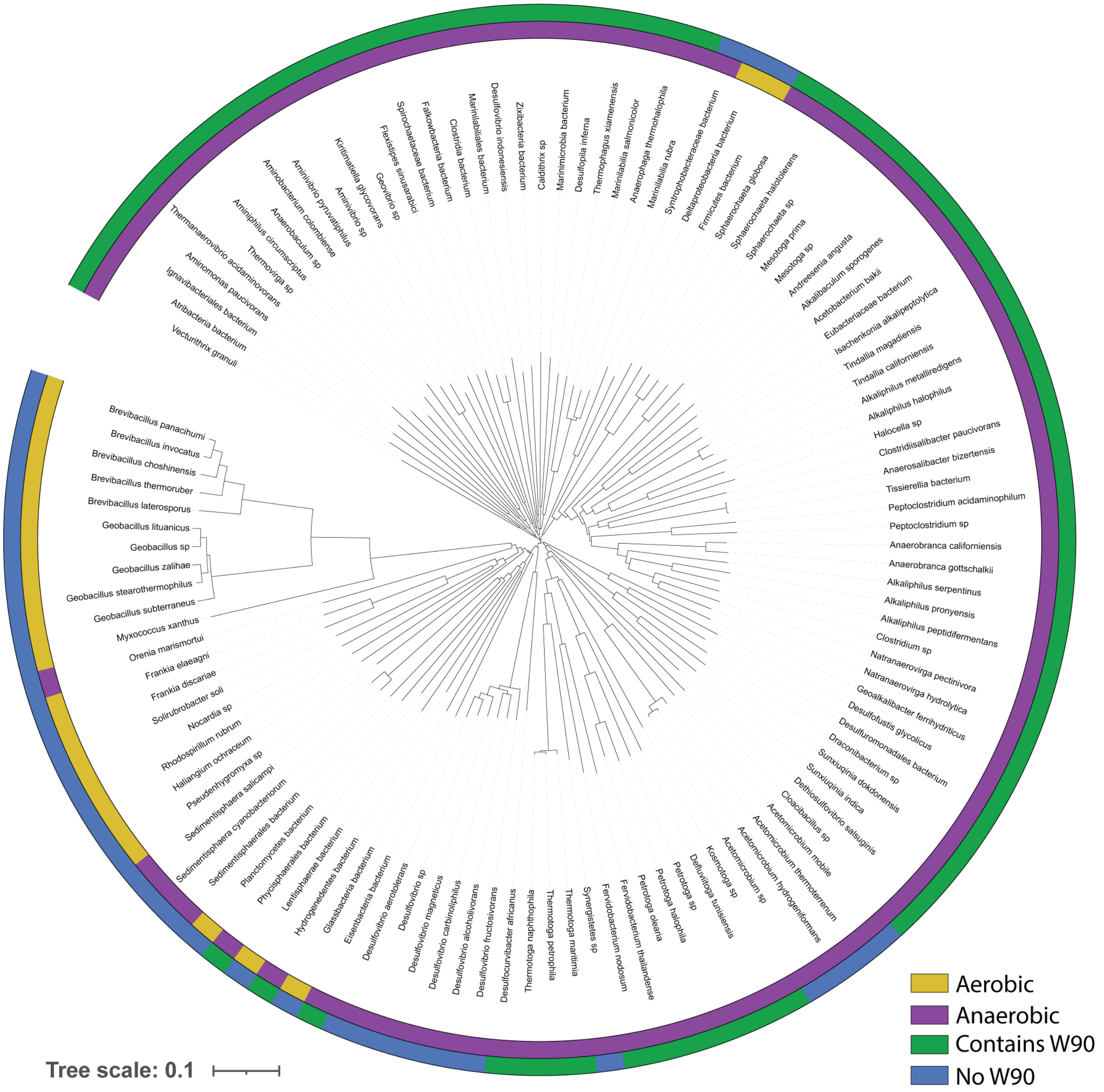

Fig. S2. Phylogenetic tree of encapsulins with FLP cargo. Phylogenetic Analysis of encapsulins related to *Thermotoga maritima*. Encapsulins that contain the W90 residue are green, with all other residues colored in blue. Encapsulins found in aerobic species are in yellow, and strict anaerobes are in purple. All W90-containing encapsulins occur in anaerobic species, however not all anaerobic species contain the W90 residue.

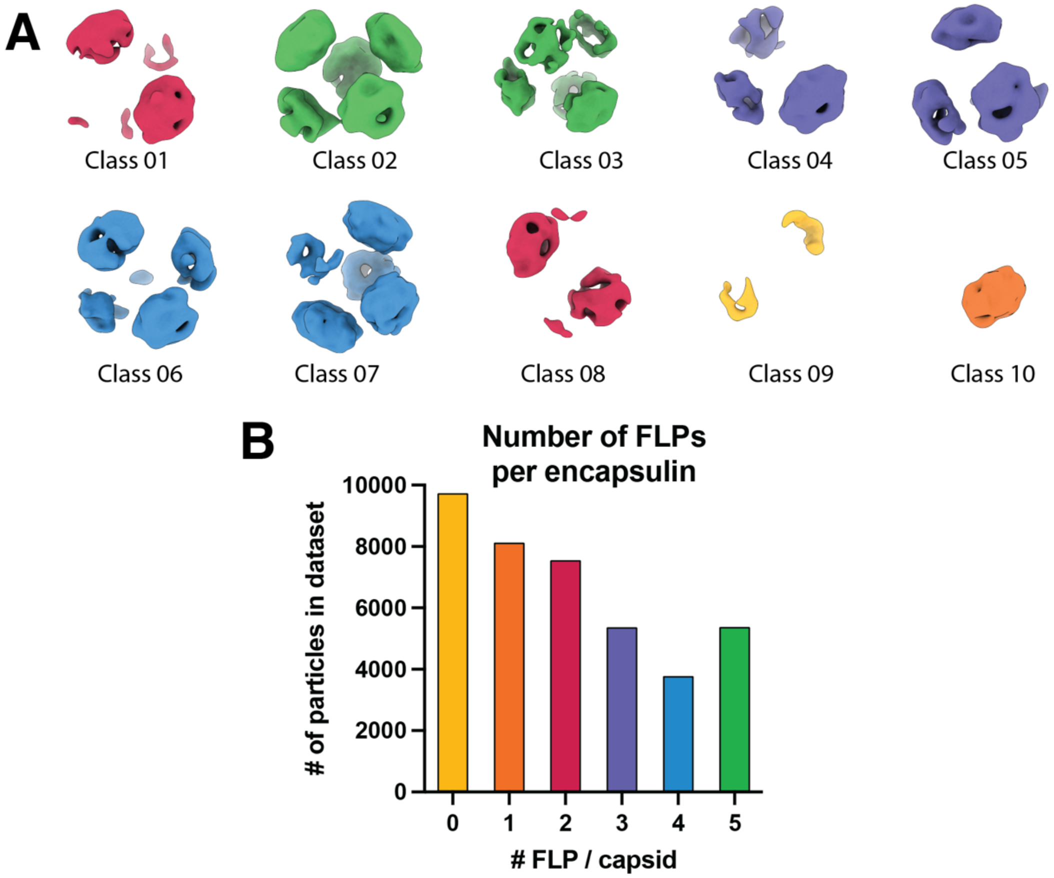

Fig. S3. Number of FLP cargo per encapsulin shell. 3D classification and analysis to determine the distribution of FLP cargo proteins per encapsulin shell. **(A)** 10 classes obtained from an alignment-free 3D classification. A mask was applied to the original C1 reconstruction to classify only the cargo FLPs. Class averages show the number of FLPs quite unambiguously. However, in rare cases where individual FLP density was weak, we compared an individual FLP density to an artificial volume created from a 10Å map of a known FLP homolog (pdb:5da5); if the weak FLP density occupied a volume less than 50% of the expected size, it was not counted. **(B)** Analysis of #FLPs/encapsulin for the dataset collected shown as a bar graph. Various loading levels from 0-5 FLPs/encapsulin were observed with a decreasing number of particles with increasing levels of loading.

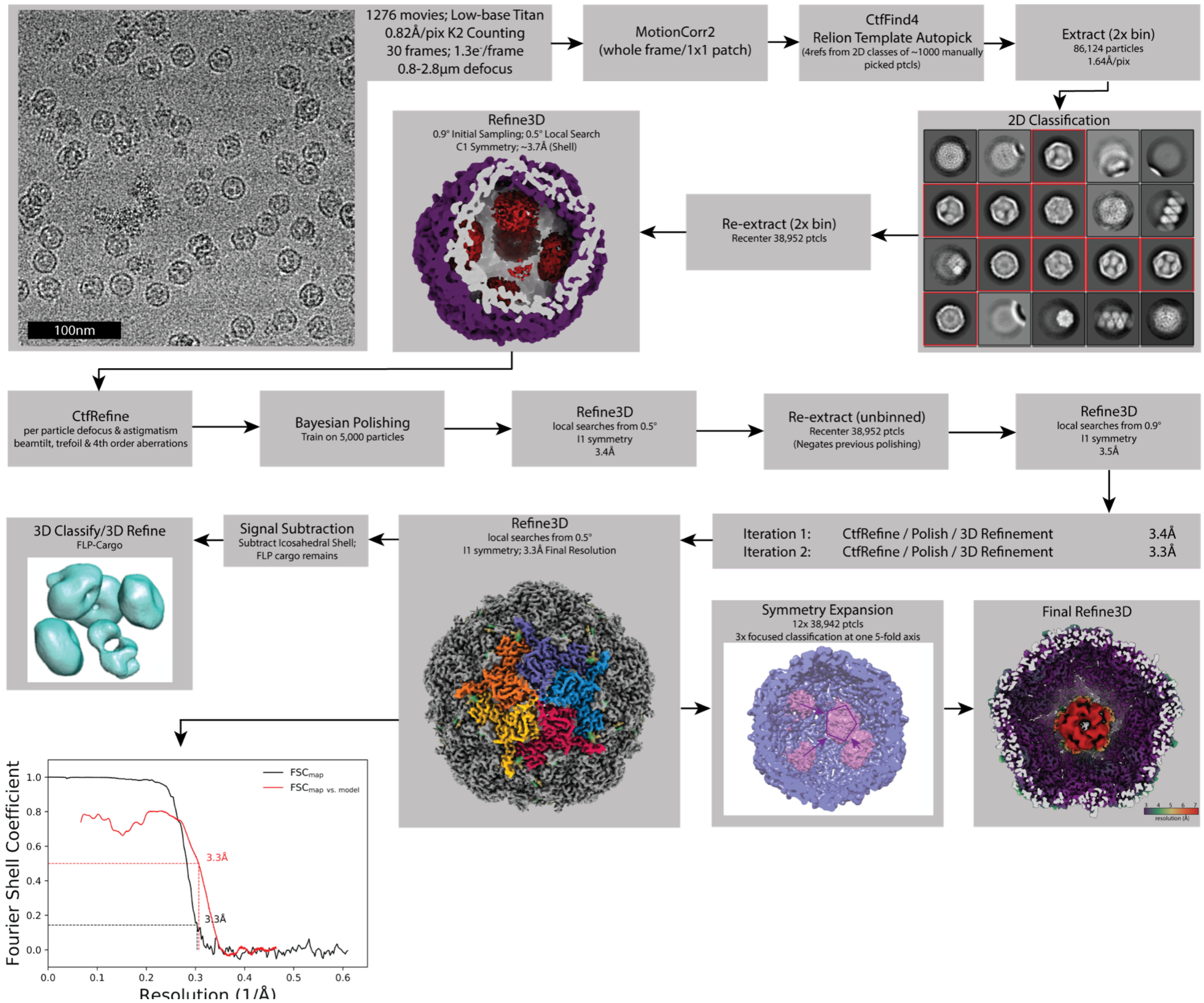

Fig. S4. Cryo-EM Processing Pipeline. Workflow used for obtaining a high-resolution encapsulin shell, as well as the symmetry expansion technique used for defining one of the possible FLP-cargo orientations relative to the shell.

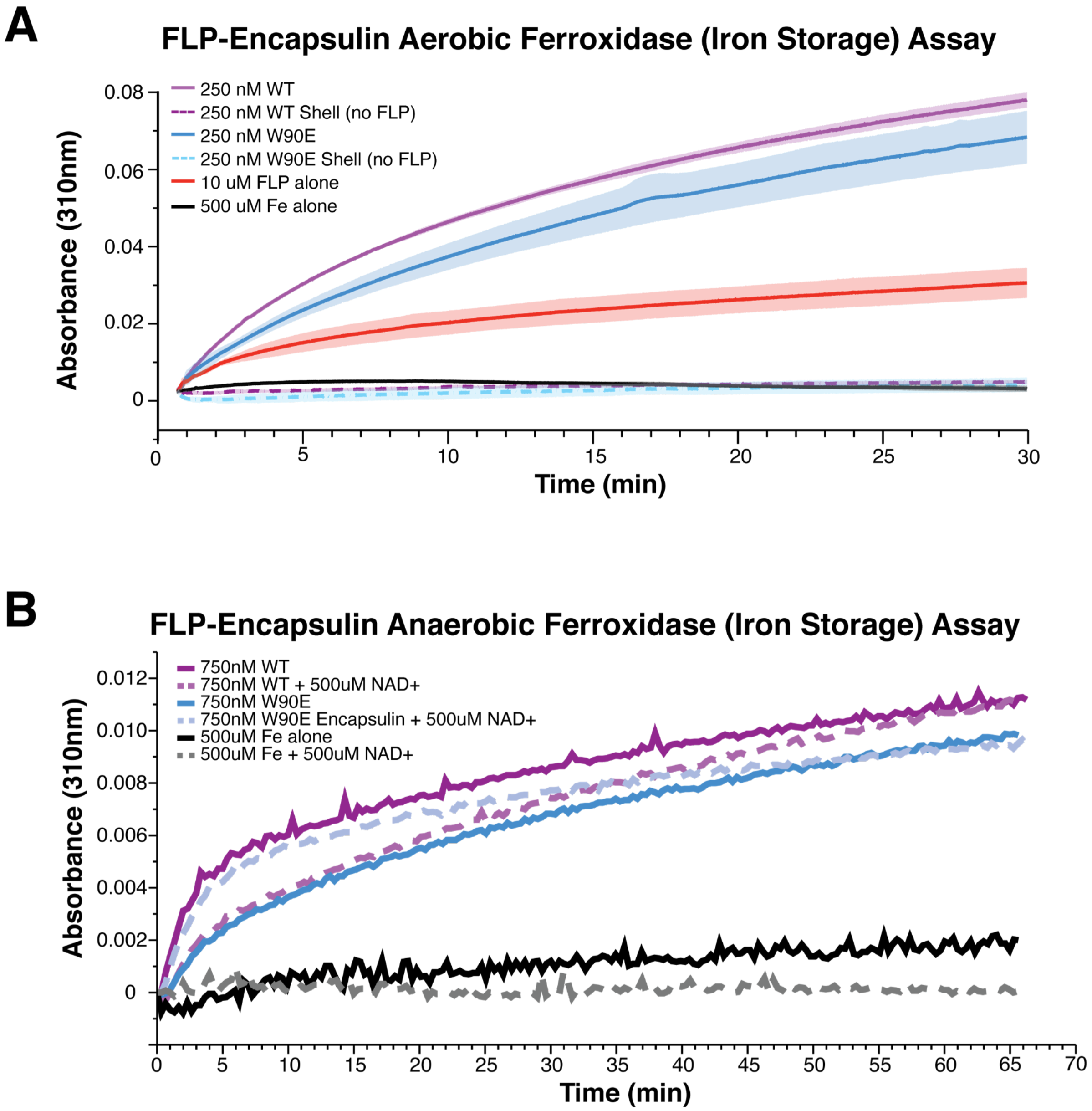

Fig. S5. The ferroxidase activity of different encapsulin constructs. All assays were performed in the presence of 500uM Fe. **(A)** Shows the ability of different encapsulins to store iron under aerobic conditions. Traces are n=3 average with the envelope representing STD. **(B)** Ferroxidase activity under anaerobic conditions. Traces represent an average of n=3. Both WT encapsulin and the W90E mutant retain the ability to store iron regardless of aerobic/anaerobic, with no significant statistical difference between the two.

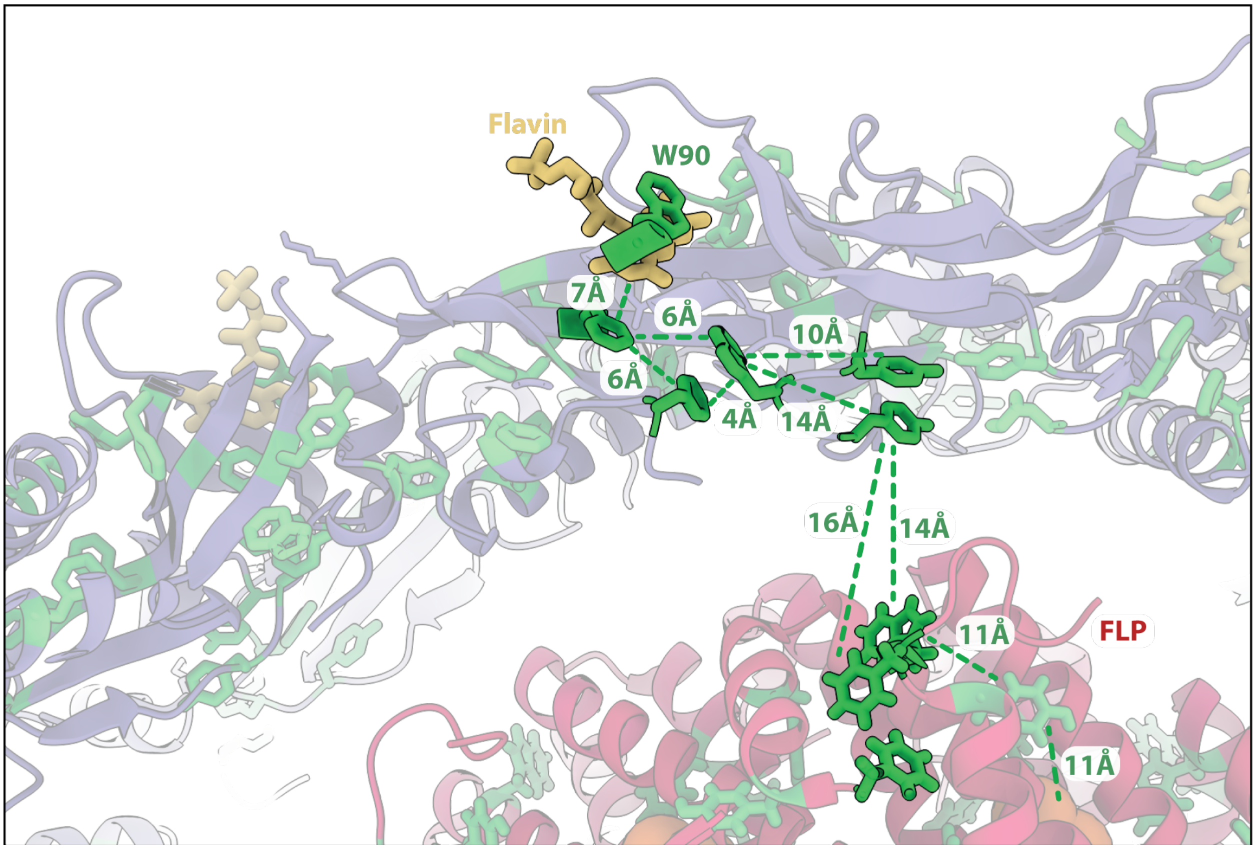

Fig. S6. Aromatics positioned between the flavin molecule and the FLP active site. Distances (in angstroms) between aromatic side chains that could serve as an electron conduit between the flavin (yellow) on the shell periphery and the active site of the encapsulated FLP cargo (orange).

**Dataset**	**Encapsulin Shell with FLP**
Microscope	Low-base Titan
Stage Type	Side-entry, Gatan 626
Voltage (kV)	300
Detector	Gatan K2
Data Collection Software	Leginon
Acquisition mode	Counting
Physical pixel size (Å)	0.82
Defocus range (μm)	0.8-2.8
Electron exposure (e^-/A^2)	40
**Reconstruction**	**EMD-24001**
Session	17Mar02
Software	RELION 3.1
Particles picked	86,124
Particles final	38,952
Extraction box size (pixels)	512^3
Final pixel size (Å)	0.82
Map resolution (Sym; Å)	3.3
Map sharpening B-factor ($Å^2$)	-150
**Coordinate refinement**	
Software	PHENIX
Refinement algorithm	REAL SPACE
Resolution cutoff (Å)	3.3
$FSC_{model-vs-map}$=0.5 (Å)	3.3
**Model**	**PDB-7MU1**
Number of residues	264
B-factor overall	38
R.M.S. deviations	
Bond lengths (Å)	0.004
Bond angles (°)	0.625
**Validation**	
Molprobity clashscore	6.68
Rotamer outliers (%)	0.0
C_β deviations (%)	0.0
Ramachandran plot	
Favored (%)	96.95
Allowed (%)	3.05
Outliers (%)	0.0

Table S1. Data collection, 3D reconstruction, and refinement statistics.
